# The association between short-term temperature variability and mortality in Virginia

**DOI:** 10.1371/journal.pone.0310545

**Published:** 2024-09-20

**Authors:** Melanie M. Pane, Robert E. Davis

**Affiliations:** Department of Environmental Sciences, University of Virginia, Charlottesville, Virginia, United States of America; United States Environmental Protection Agency, UNITED STATES OF AMERICA

## Abstract

The objective of this study is to determine the relationship between short-term temperature variability on neighboring days and mortality. The change in maximum temperature in Northern Virginia, Richmond, Roanoke, and Norfolk, Virginia, on neighboring days was calculated from airport observations and associated with total mortality over a multi-county area surrounding each weather station. The association between day-to-day temperature change and mortality, lagged over a 28-day period, was analyzed using distributed lag non-linear models that controlled for air quality, temporal trends, and other factors. Days following large temperature declines were associated with an increased risk of mortality in three of the four locations, and temperature increases were linked to higher mortality risk in two cities. For example, the relative risk of mortality for a 12°C daily temperature decline (1^st^ percentile) was 1.74 [0.92, 3.27] in Roanoke and 1.16 [0.70, 1.92] in Richmond. The net effect of short-term temperature increases was smaller, with the largest relative risk of 1.03 [0.58, 1.83] for a 12°C increase (99^th^ percentile) in maximum temperature in Norfolk. In Richmond and Roanoke, there was an observed lagged effect of increased mortality (maximum relative risks varying from 1.08 to 1.10) that extended from 5 to 25 days associated with large temperature declines of 15°C or more. In contrast, there was a strong and immediate (lag 0–3 day) increase in the risk of mortality (1.10 to 1.15) in northern Virginia and Norfolk when the temperature increase exceeded 10°C (short-term warming). In general, consecutive day warming had a more immediate mortality impact than short-term cooling, when the peak mortality is lagged by one week or more. However, cooling of at least 10°C after a hot (summer) day reduced mortality relative to comparable cooling following a cold (winter) day, which is associated with high mortality. This differential mortality response as a function of temperature suggests that there is some relationship between average temperature, temperature variability, and season. The findings of this study may be useful to public health officials in developing mitigation strategies to reduce the adverse health risks associated with short-term temperature variability.

## Introduction

There is a widespread consensus that human health is sensitive to weather and climate. Previous studies have found that high temperatures and heat waves as well as low temperatures and cold waves are associated with a multitude of adverse health outcomes, including mortality [1–4]. A related issue is the role of short-term weather changes on health, with the associated implication that lack of acclimatization to changing environmental conditions can enhance physiological strain on the body [5]. Individuals with preexisting conditions and the elderly population are at higher risk of being severely impacted by abrupt changes in temperature [6–9]. Thus, better understanding of the effects of temperature variability on human health and mortality is crucial to improving public health practices, policies, and interventions that would minimize the resulting health consequences.

Temperature and mortality relationships vary widely depending on the climate and region of interest, but are generally characterized by J, V, W, and U-shaped associations [10]. Cold effects tend to peak after a short lag and persist for several weeks whereas heat effects are more likely to have an immediate impact and last for only a few days [11]. Short-term temperature variations can be examined from both intra-day and inter-day standpoints. The diurnal temperature range, or the difference between the daily maximum and minimum temperature, is one commonly used measure of short-term temperature variability [9, 12]. Temperature change between neighboring days, based on the difference in maximum or mean temperatures, is frequently used to examine inter-day temperature variation. Large variations in temperature on consecutive days have been associated with a person’s heart rate, blood pressure, blood cholesterol levels, plasma fibrinogen concentrations, peripheral vasoconstriction, platelet viscosity, autonomic control of the heart, and the immune system’s capability to resist infections [9, 13].

The goal of this study is to determine whether large changes in temperature between neighboring days have an impact on mortality. Four locations in Virginia—Northern Virginia (suburban Washington, D.C.), Richmond, Roanoke, and Norfolk—are used to examine this potential association. A related goal is to determine if the mortality response to temperature change is independent of temperature—whether the initial temperature prior to the rapid rise or drop is related to mortality counts.

## Materials and methods

### Weather data

**Fig 1:**
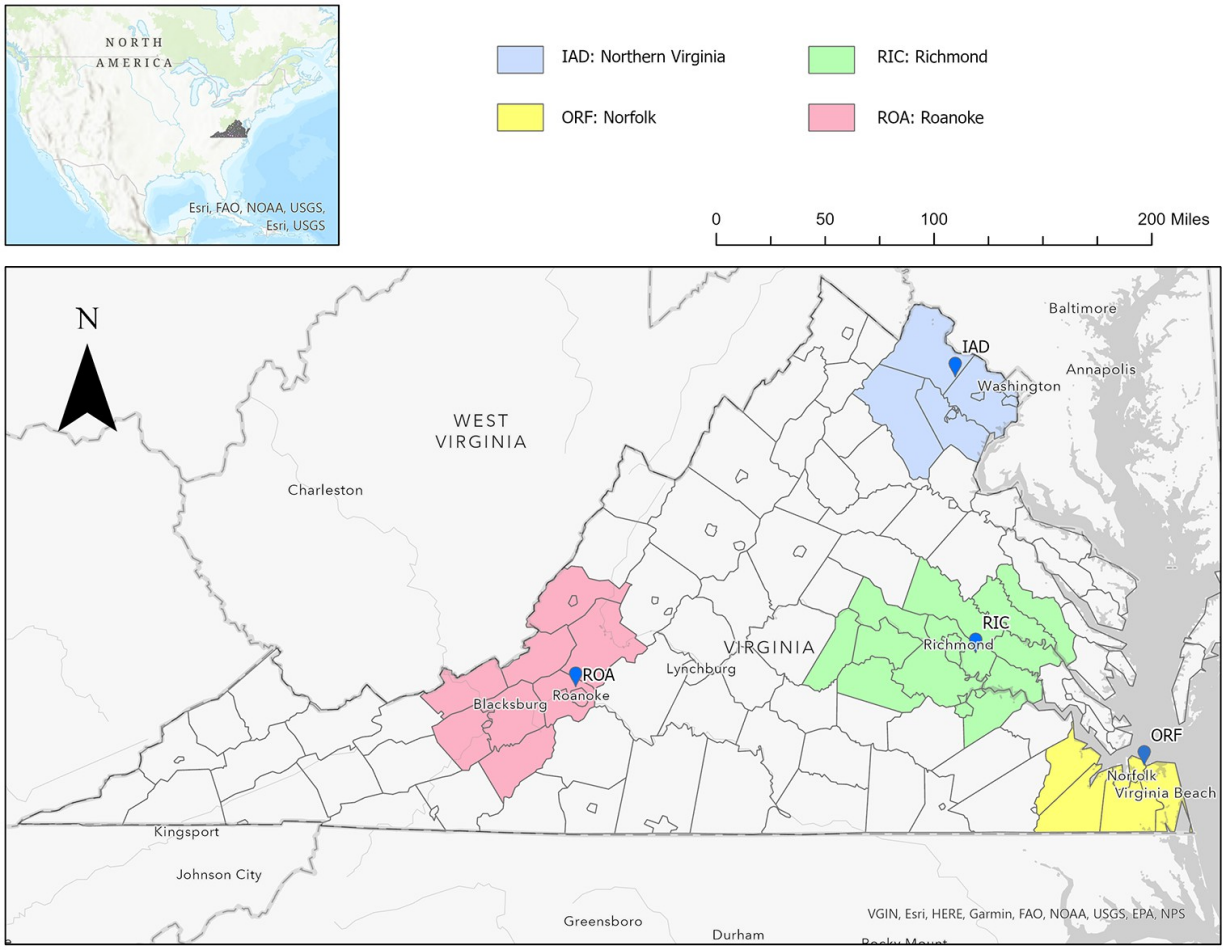
Map of the counties and independent cities representing the four Virginia regions used in this study: IAD, RIC, ROA, ORF. Base map from ESRI (VGIN, Esri, HERE, Garmin, FAO, NOAA, USGS, EPA, NPS; url: https://www.arcgis.com/home/item.html?id=979c6cc89af9449cbeb5342a439c6a76).

Weather data were collected from four airport weather stations across Virginia: Northern Virginia (IAD–Dulles International Airport), Richmond (RIC–Richmond International Airport), Norfolk (ORF–Norfolk International Airport), and Roanoke (ROA–Roanoke/Blacksburg Regional Airport). These four locations were chosen from the most populated areas in the Commonwealth of Virginia. Each of the weather stations is used to represent the climate of a larger area of surrounding counties and independent cities (Fig 1 and S1 Table). Weather data were downloaded from the Iowa State University Environmental Mesonet website (https://mesonet.agron.iastate.edu/) for the period from January 1, 2005 to December 31, 2020. Temperature was collected at 1 a.m., 7 a.m., 1 p.m., and 7 p.m., or the next closest available observation time. If a singular observation was missing, the missing data point was linearly interpolated using the neighboring times. If more than one observation was missing, the values were coded as missing.

A daily mean temperature was calculated by computing the mean of the four daily temperature observations. That variable was then used to calculate the mean temperature change between neighboring days by subtracting the current day’s mean temperature from the previous day’s mean temperature [6, 9, 14]. A positive value represents an increase in temperature between neighboring days, whereas a negative value represents a decrease in temperature between days. Daily values of maximum temperature and minimum temperature were acquired from the National Oceanic and Atmospheric Administration Summary-of-the-Day compilations over the same time period. With these values, we calculated the maximum temperature change by subtracting the current day’s maximum temperature from the previous day’s maximum temperature [8].

Daily air quality data for concentrations of ozone and particulate matter with a diameter smaller than 2.5 microns (PM_2.5_) were accessed from monitors in each city from the U.S. Environmental Protection Agency archives (EPA—https://www.epa.gov/outdoor-air-quality-data/download-daily-data). The daily mean values of all available PM_2.5_ observations and the maximum daytime (8 h) ozone concentrations were used in our analysis. Each region typically contained multiple air quality monitoring stations, so for each observation we calculated a z-score, for both ozone and PM_2.5_, using the mean and standard deviation from each station. Then, we computed the mean z-score per day using all available monitors to obtain a regional average. We utilized this z-scoring approach to mitigate against the possibility that a single monitoring station sited in a location with poor air quality could disproportionately skew the mean values. This approach allowed us to make use of all the available air quality data while emphasizing the daily change in air quality rather than the absolute concentrations, as the within-region variability is more important in building our models.

### Mortality data

Patient-level mortality data were acquired from the Interjurisdictional Exchange Mortality File from the Virginia Department of Health, Office of Information Management. These files use death certificate records and include detailed information on the decedent, including date of death and county (or independent city) of residence. This is the complete population of Virginia mortality and is not a sample. A mortality case is counted based on the county/city of residence, and total daily mortality was then summed for each of the four regions from 2005–2020. This project was determined to be "non-human subject research" by the Institutional Review Boards of the Virginia Department of Health and the University of Virginia.

### Data analysis

Distributed lag non-linear models (DLNMs) were used to investigate the magnitude of the effect of short-term variations in temperature on mortality [e.g., 4, 6–8, 12, 14, 15]. These models utilize a time-series based approach to identify the relationship between potential predictor variables (weather variables) and the outcome or response variable (mortality). DLNMs allow for the examination of the temporal dynamics of that relationship in lag space, i.e., how mortality varies as a function of both weather variables and lag.

We initially used mean temperature change and maximum temperature change as estimates of short-term thermal variability [6, 8, 9, 14]. Upon testing these two temperature variables, we found that maximum temperature change produced models with a slightly better fit to our data based on the generalized cross validation (GCV) coefficient, Akaike’s Information Criterion (AIC), and the deviance explained (S2 Table).

The final model used in this study is as follows:

$$Log(Y_{t})=a+S\left(MaxTempDiff,3\right)+S\left(Trend,16\times 3\right)+S(MeanTemp,3)+S\left(Ozone,3\right)+S\left(PM2.5,3\right)+dDOW_{t}$$ (1)

where *Y* is the daily mortality count, *t* is the time counter (in days), *a* is the y-intercept, *MaxTempDiff* is the maximum daily temperature change with 3 degrees of freedom, *S* is a natural cubic spline, *MeanTemp* is the daily average temperature with 3 degrees of freedom, *Trend* is a term that accounts for long-term temporal variations in the response variable with 3 degrees of freedom used for each of the 16 years, *Ozone* is the z-score of the ozone levels with 3 degrees of freedom, *PM*_*2*,*5*_ is the z-score of the particulate matter levels with 3 degrees of freedom, and *DOW* is a nominal variable for day of the week. Controlling for the day of the week in the model is a common practice for investigating the potential for weekend versus weekday effects [8, 14]. A quasi-Poisson log link function was utilized to account for the overdispersion that is typically associated with count data. Each model was tested for overdispersion, and in cases where overdispersion was not evident, a Poisson link was employed. Polynomial splines with 1 knot were utilized to fit the predictor variables while natural cubic splines with 2 knots in natural log space were fitted for the lag dimension. DLNM lags were run through 28 days after comparison to a 21-day lag model, and 0ºC (no change) was the temperature used to center the relative risk (RR) estimates. The final model presented here was selected after testing a variety of different predictor variables, including relative humidity and diurnal temperature range. Final models were selected based on the generalized cross validation coefficient and deviance explained, and all predictors were examined for significance. We also conducted tests by varying the degrees of freedom in the trend term, the number and locations of knots, and the types of splines used in fitting the data. These comparisons are summarized in S2 and S3 Tables and S1–S3 Figs.

In addition to examining the impact of daily temperature variability on mortality, we explored possible relationships as a function of initial temperature/season. We wished to determine if, for example, a temperature change of 10ºC between consecutive days would have the same effect on mortality at 30ºC as it would at 0ºC. To address this question, the data for each region were first divided into two subsets. The first subset included all days with maximum temperature increases ≥ 10ºC, and the second subset included all days with maximum temperature declines ≥ 10ºC. Each day in these subsets was then assigned to the appropriate 5ºC wide temperature bin, based on the first day’s temperature, and one-way analysis of variance (ANOVA) was used to compare mean mortality across bins. A significance level of 0.05 was used for the ANOVA to compare means across multiple groups, followed by the Tukey *post hoc* test to identify those groups with statistically significant differences. A 10°C temperature change threshold was selected after testing other values (5°C and 15°C) and considerations for a balance between the total number of bins and having enough available samples within each bin for statistical robustness. Each bin consisted of a minimum of 10 observations, which in some cases necessitated combining the highest and lowest bins with adjacent bins. Therefore, the maximum temperature bin is 15°C as there were insufficient observations at higher values to produce a robust sample.

## Results

**Fig 2:**
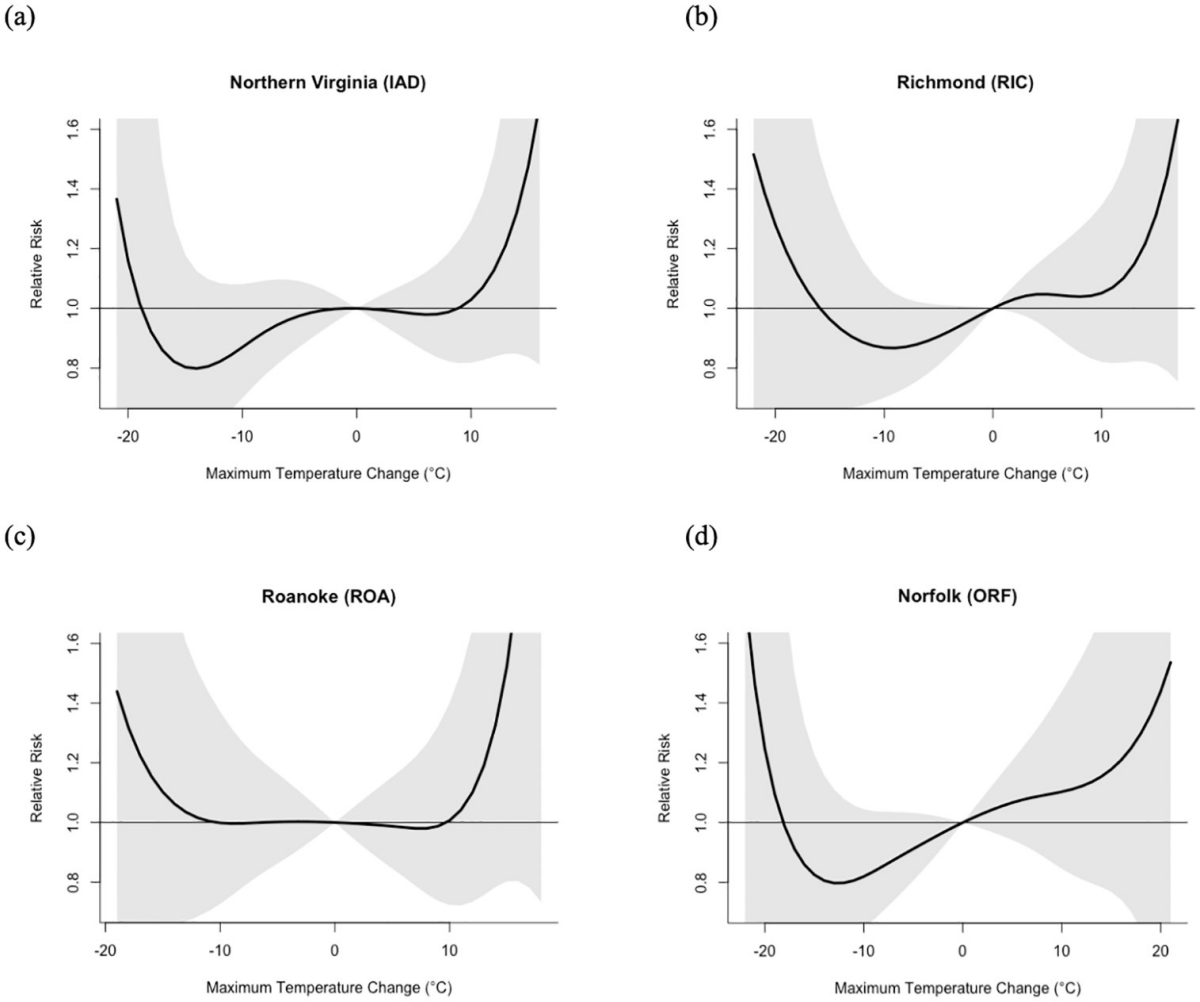
Overall cumulative association between maximum temperature change and relative risk of mortality for (a) Northern Virginia, (b) Richmond, (c) Roanoke, and (d) Norfolk. The gray shading indicates two standard errors above and below the relative risk. These estimates include a 28-day lag period.

We find a U- or J-shaped relationship between mortality and short-term temperature variability between neighboring days. The analysis of the data for all four study locations—Northern Virginia, Richmond, Roanoke, and Norfolk—shows that both large daily maximum temperature increases and decreases are associated with an increased RR of mortality over a 28-day lag period, with the overall pattern varying between locations (Fig 2 and Table 1).

**Table 1:** Relative risk (RR) of mortality as a function of temperature change (dT) percentile for each location. Confidence intervals (95%) are shown in brackets.

Station	dT	RR	dT	RR	dT	RR	dT	RR	dT	RR
	1%	1%	5%	5%	50%	50%	95%	95%	99%	99%
IAD	–12°C	0.91 [0.58, 1.43]	–8°C	0.98 [0.72, 1.33]	0°C	1.00	7°C	0.89 [0.68, 1.17]	11°C	0.90 [0.58, 1.38]
RIC	–12°C	1.16 [0.70, 1.92]	–8°C	1.06 [0.76, 1.49]	1°C	0.98	7°C	0.83 [0.62, 1.13]	11°C	0.78 [0.49, 1.25]
ORF	–13°C	0.83 [0.45, 1.53]	–8°C	0.85 [0.58, 1.23]	1°C	1.00	8°C	0.97 [0.65, 1.43]	12°C	1.03 [0.58, 1.83]
ROA	–12°C	1.74 [0.92, 3.27]	–8°C	1.41 [0.93, 2.13]	0°C	1.00	7°C	0.72 [0.50, 1.04]	12°C	0.77 [0.40, 1.46]

In general, temperature declines tend to have a greater impact on mortality than temperature increases. In Roanoke and Richmond, the highest mortality RR is associated with temperature changes that exceed –10 to–15°C, respectively. For a temperature change of –12°C (1^st^ percentile), the RR is 1.74 [0.92, 3.27] in Roanoke and 1.16 (0.70, 1.92) in Richmond. In contrast, there is no effect of declining temperatures evident in Northern Virginia, and the U-shape in Norfolk is deceptive given that the mortality RR is well below 1.0 at the 1^st^ percentile of temperature change (–13°C), so the very high mortality risk for extremely large temperature drops is based on very rare events and only a few data points. In contrast, Norfolk does exhibit a mortality increase at the 99^th^ percentile of maximum temperature change (+12°C), but the RR of 1.03 [0.58, 1.83] is comparatively low. Roanoke also exhibits increased mortality for temperature changes that exceed +15°C, but these events are rare, and the robustness of the model fit is tenuous given the small sample size.

**Fig 3:**
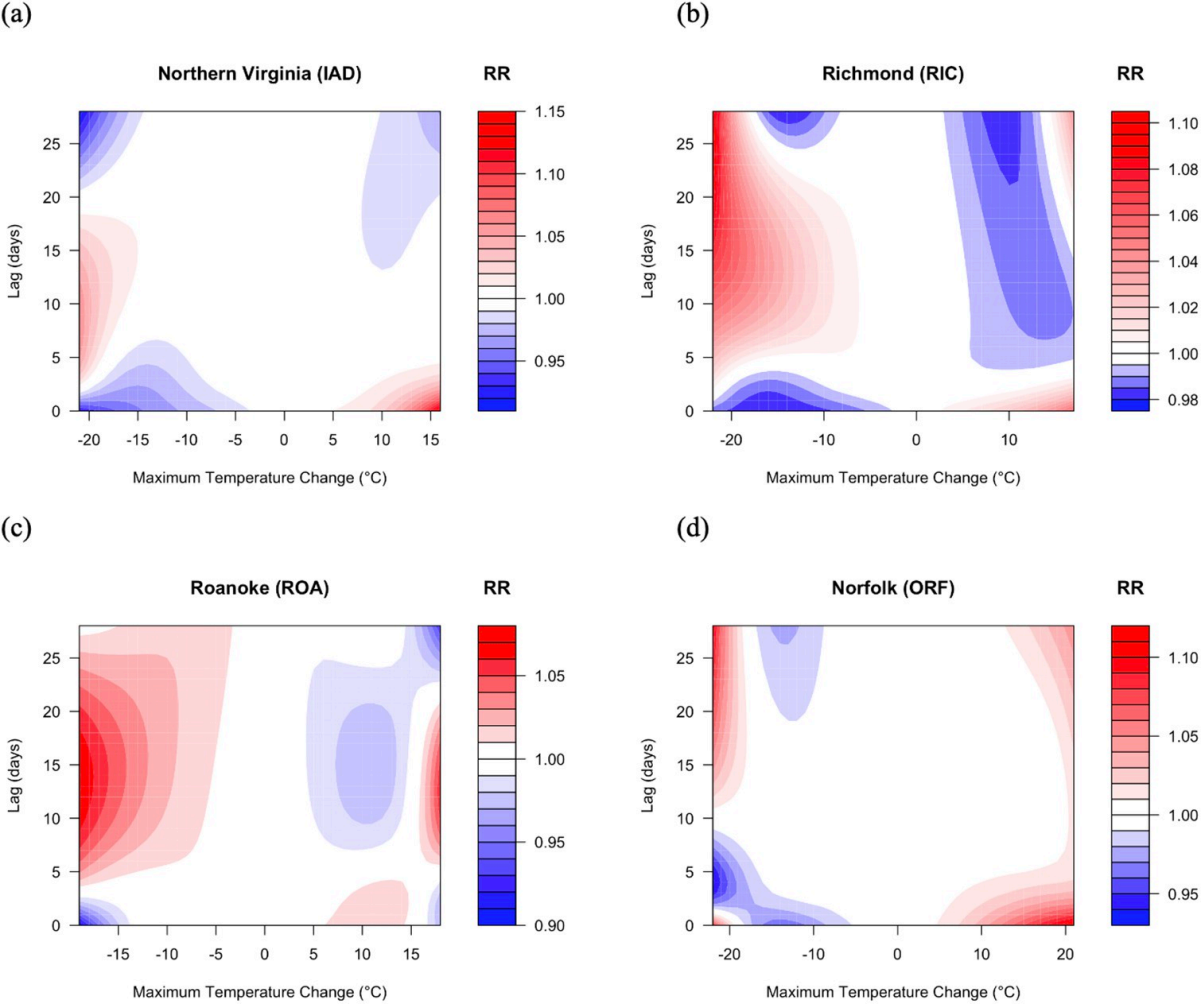
Relative risk of mortality as a function of maximum temperature change and lag for (a) Northern Virginia, (b) Richmond, (c) Roanoke, and (d) Norfolk.

Examination of the lagged effects reveals a consistent pattern across all study locations. In each region, there is a lagged effect of high mortality occurring some number of days after an initial extreme temperature decrease. Northern Virginia initially exhibits a protective effect at moderately and extremely negative temperature changes, followed by a 5–10% increased risk of mortality 5 to 18 days later (Fig 3a). Richmond shows a similar initial protective effect until day 3, followed by a 2–8% increased risk of mortality from lag 5 onward (Fig 3b). Roanoke also exhibits a protective effect to about 4 days, but its lagged effect extends from 6 to 25 days with an increased risk of 4–10% (Fig 3c). In contrast, Norfolk has the longest protective effect period of around 6 days, followed by a lagged effect of 2–5% on days 10 to 25 (Fig 3d).

An immediate and strong increase in the risk of mortality is observed in association with warming, particularly for very large temperature increases. This increased risk of mortality is strongest in Northern Virginia (e.g., RR = 1.10 [1.01, 1.20] at +16°C and lag 0) and Norfolk, with a maximum RR of 1.16 [1.01, 1.33] that occurs at lag 0 (Fig 3a and 3d, respectively). Richmond displays a smaller increased risk of 1.03 [0.98, 1.07] at lag 0, whereas Roanoke shows its highest increased risk of 1.02 [0.93, 1.12] after lag 4 (Fig 3b and 3c, respectively).

**Fig 4:**
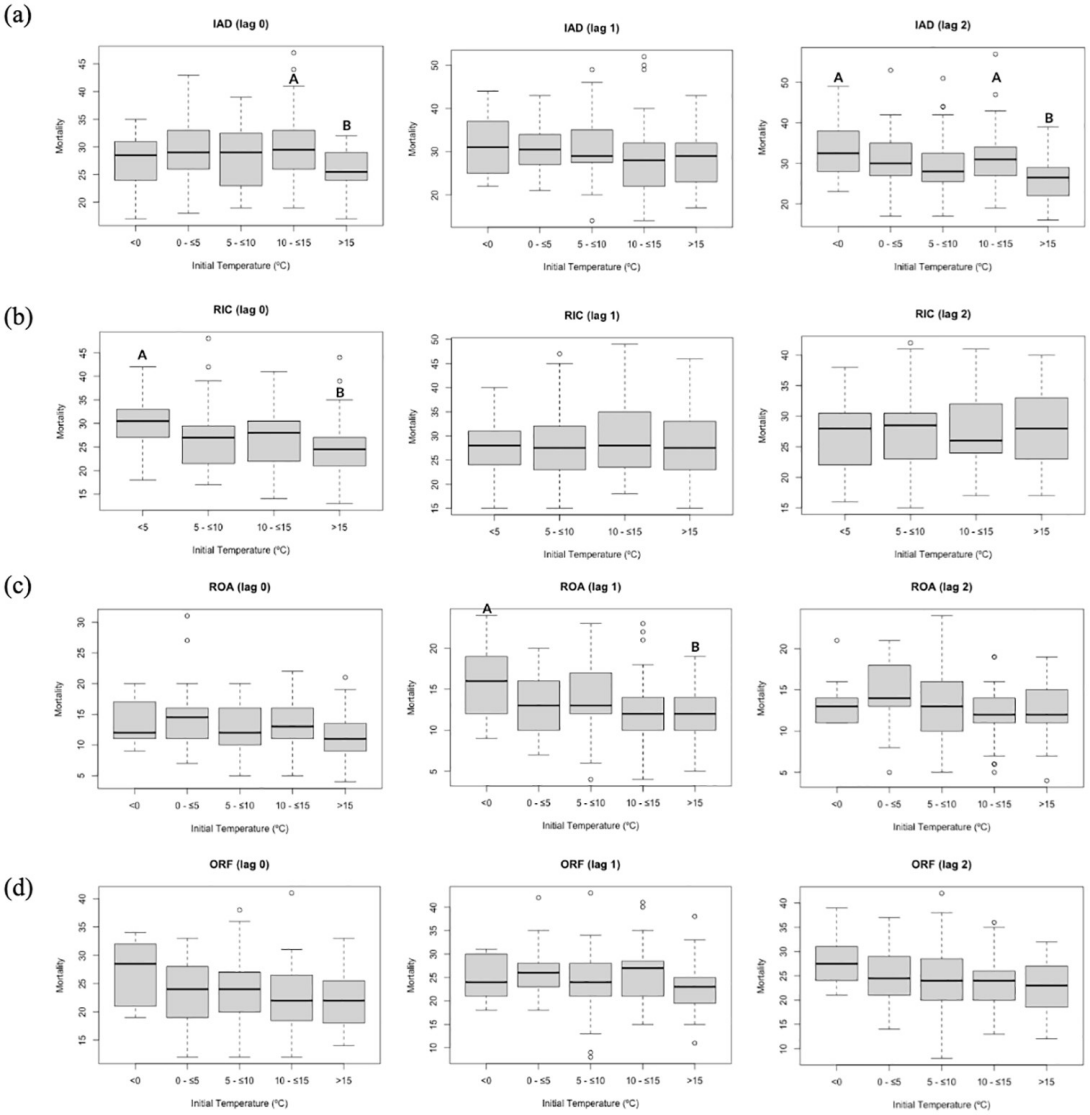
Mortality on days when maximum temperature declined by at least 10°C as a function of initial (Day 1) temperature at Lag 0, 1, and 2 for (a) Northern Virginia, (b) Richmond, (c) Roanoke, and (d) Norfolk. Mortality in bin A is significantly different than bin B based on the Tukey *post hoc* test (p < 0.05) for those cases with overall significant differences based on the analysis of variance.

Next, we examined if the starting temperature on the day prior to the large temperature change was related to mortality differences. In general, the overall pattern shows a decline in mean bin mortality with increasing temperature (Fig 4). In other words, strong cooling after a hot day is less harmful than strong cooling following a cold day. Statistically significant differences are found between the warmest and coldest bins in Richmond (lag 0), Roanoke (lag 1), and Northern Virginia (lag 2). Northern Virginia also exhibited a significant mortality difference between the warmest and next warmest bins (lags 0 and 2). Although *post-hoc* tests found no significant differences in Norfolk, the overall trend is consistent with the other three locations. These results suggest that, at least with respect to cooling, there is some association between temperature and consecutive day temperature variability.

**Fig 5:**
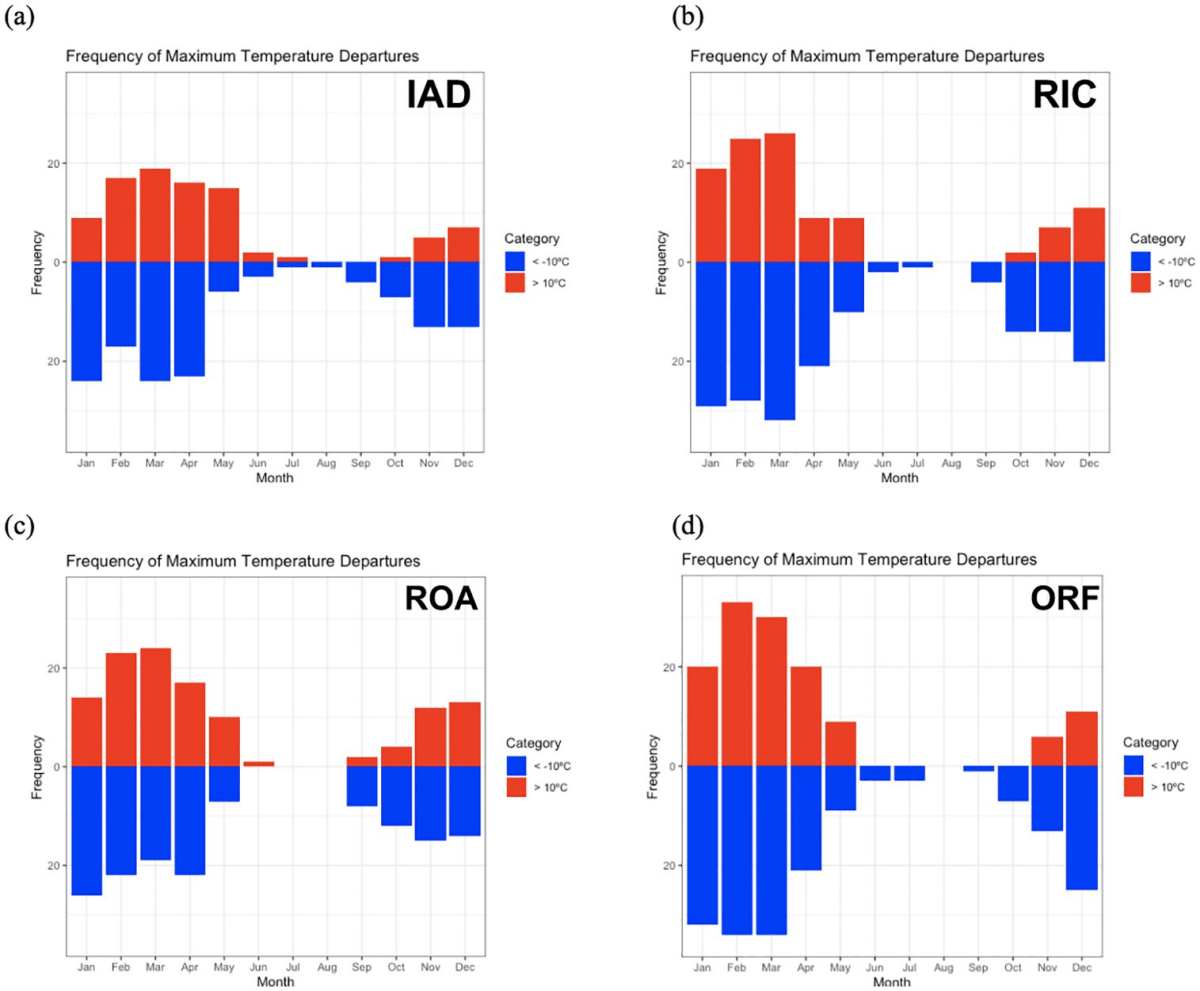
Frequency of both positive and negative maximum temperature changes ≥10°C between neighboring days by month from 2005–2020 for (a) Northern Virginia, (b) Richmond, (c) Roanoke, and (d) Norfolk.

The climatology of 10°C daily temperature changes show a clear prevalence for these events in the cold season (October–March) (Fig 5). This is dynamically consistent with the cold-season proximity of the polar front jet and associated frontal passages. Large day-to-day temperature changes become less common in the warm season as the tropospheric polar vortex weakens and retreats poleward, and along with it the associated cyclones and fronts. In general, large temperature drops, most often related to cold front passages, are more common than rapid temperature increases. This is consistent with frontal cyclone observations, as warming after a frontal passage tends to be more gradual as the warm-core anticyclone gradually migrates from west to east and the winds become more southerly prior to the next cold front passage [16, 17]. The prevalence for temperature declines over increases is most prominent in the autumn as the intensity of cold, polar air masses increases in conjunction with seasonal north polar cooling. Conversely, in May, daily increases are more common, but the difference is much more obvious in the autumn.

## Discussion

The relative risk of mortality exhibits a J-shaped or U-shaped relationship to daily temperature change, with both large temperature increases and decreases associated with elevated death counts in the four regions of Virginia examined here. In general, large short-term temperature declines exact a greater mortality toll than temperature increases, but this response is not evident in Northern Virginia, the locale with the largest population. These results are generally consistent with prior research conducted on populations in Australia [14], China [6, 8], and the United States [9]. Our findings are likewise consistent with studies showing an acute impact from strong warming, but a longer lagged effect associated with short-term cooling [3, 4 6, 11].

A physiological understanding of the reasons for these mortality increases remains elusive. These relationships imply that some vulnerable individuals lack the capacity to adapt to short-term changes in their thermal environment, thus representing a lack of acclimatization [18, 19]. The reduced sweating response among the elderly and very young is well known [e.g., 20], but it is not clear how a short-term temperature increase that might induce this response differs in its imposition of stress on the body compared to a gradual temperature increase [21–24]. Abrupt cooling has been examined in the context of physiological strain imposed on the respiratory system [25, 26]. A few studies have demonstrated that short-term cooling is associated with increased respiratory emergency department admissions and mortality [27, 28], which could be associated with the lack of acclimatization to cold and often dry conditions. Our temperature bin analysis, which generally shows that strong day-to-day cooling is linked to higher mortality than warming, supports this research. However, a more detailed examination of the subset of people who die on these days with short-term weather changes could elucidate these relationships, but that analysis is beyond the scope of this study.

The analysis of temperature bins suggests that the relationship between temperature change on neighboring days and mortality is influenced by season. In particular, the results indicate that short-term temperature declines have a greater impact on mortality in the winter months, when there are greater temperature fluctuations, compared to the months of June through September. Some prior researchers found greater impacts of temperature variability in the cool season [8, 29], whereas others detected larger mortality in the warm season [9, 10]. Furthermore, a multi-country study reported that the effect estimates for temperature variability and mortality associations were higher during the moderate/transitional seasons compared to the hot and cold seasons in 10 of the 12 countries examined [13]. Differences in methods, climates, and the underlying populations make it difficult to compare results across studies. Thus, there remains a need for future research to specifically address the season-specific aspects of daily temperature variability and health.

The increased mortality risk during the colder months could be attributed to the nature of cold front passages, in which temperature declines are typically more sudden and abrupt, as opposed to warm front passages, which tend to be associated with more gradual warming. We found no relationship between initial temperature and mortality for temperature increases. These relationships may also be impacted by the inherent seasonality in mortality, which typically peaks in January in Virginia [4]. This is a topic for future research.

### Limitations

The results of this study of four Virginia regions may not be generalizable to other populations or locations. However, given that similar U-shaped relationships have been uncovered between mortality and short-term temperature change in other mid-latitude locations with temperate and variable climates, we believe our results are consistent with prior, related research. U-shaped relationships imply that the greatest risk is at the edges of the distribution which are based on weather events that are relatively rare; thus the estimates at the extremes are more error-prone. We therefore have emphasized the general pattern moreso than the specific risk estimates. Additionally, although Roanoke is one of the largest cities in the western part of the state, it is still not as large of a metropolitan area as Richmond, Northern Virginia, or Norfolk. Thus, Roanoke’s daily sample size is the smallest of the four locations and may be subject to more daily fluctuations. Other methods have been proposed to characterize day-to-day temperature variability [30]. Although our method is simple and has been used by other researchers, it may not have the strongest underlying relationship to mortality.

## Conclusions

We find statistically significant associations between short-term temperature variability and mortality in Northern Virginia, Richmond, Roanoke, and Norfolk. Specifically, large temperature changes between neighboring days, both positive and negative, are coupled with increased mortality, but the magnitude of the effects varies significantly across locations. In all regions, a lagged effect was observed when the temperature change was extremely negative and an immediate and strong increase in the risk of mortality was observed for extremely positive temperature changes.

Further research is warranted to examine possible physiological linkages to short-term thermal changes. We hope to exploit the availability of patient-level diagnostic data in future analyses. Overall, our findings underscore the impact of short-term temperature variability on mortality and suggest that further research is needed to explore how public health interventions can be used to mitigate the adverse health impacts that occur in association with short-term temperature changes. Given that these changes can often be predicted with accuracy days in advance, this information could be useful, particularly if the most vulnerable at-risk populations are identified (based on patient demographics and specific disease characteristics, etc.).

## Supporting information

S1 Table List of counties and independent cities associated with each of the four regions examined: Northern Virginia (IAD), Norfolk (ORF), Richmond (RIC), and Roanoke (ROA). (DOCX)

S2 Table Generalized additive model diagnostic information for the maximum and mean temperature difference models. Dof: degrees of freedom, dow: day of week, GCV: generalized cross validation, AIC: Akaike’s Information Criterion.
(DOCX)

S3 Table Akaike’s Information Criterion (AIC) for varying numbers of equally-spaced knots and types of splines for maximum temperature difference. The base model is summarized in Eq 1. ns: natural cubic spline, bs: polynomial spline.
(DOCX)

S1 Fig Fit of the trend term only for IAD. The number at the top of each figure indicates the degrees of freedom per year. (Note that with more than 3 degrees of freedom, there is little change in the overall pattern).
(DOCX)

S2 Fig Comparison of DLNM results for a maximum lag of 21 vs. 28 days at RIC and ORF. (DOCX)

S3 Fig Impact of number of equally-spaced knots or overall relative risk estimates as a function of maximum temperature change at ROA. 4 knots (top left), 5 knots (top right), 6 knots (bottom left), 7 knots (bottom right).
(DOCX)

## References

[1] Basu R. High ambient temperature and mortality: a review of epidemiologic studies from 2001 to 2008. Environmental Health. 2009 Dec;8:1–3. doi: 10.1186/1476-069x-8-40 19758453 PMC2759912

[2] Gasparrini A, Armstrong B, Kovats S, Wilkinson P. The effect of high temperatures on cause-specific mortality in England and Wales. Occupational and Environmental Medicine. 2012 Jan 1;69(1):56–61. doi: 10.1136/oem.2010.059782 21389012

[3] Ye X, Wolff R, Yu W, Vaneckova P, Pan X, Tong S. Ambient temperature and morbidity: a review of epidemiological evidence. Environmental Health Perspectives. 2012 Jan;120(1):19–28. doi: 10.1289/ehp.1003198 21824855 PMC3261930

[4] Davis RE, Roney PC, Pane MM, Johnson MC, Leigh HV, Basener W, et al. Climate and human mortality in Virginia, 2005–2020. Science of The Total Environment. 2023 Jun 19:164825. doi: 10.1016/j.scitotenv.2023.164825 37343846

[5] De Freitas C.R., Grigorieva E.A. The Acclimatization Thermal Strain Index (ATSI): a preliminary study of the methodology applied to climatic conditions of the Russian Far East. Int J Biometeorol 53, 307–315 (2009). doi: 10.1007/s00484-009-0215-6 19238456

[6] Lin H, Zhang Y, Xu Y, Xu X, Liu T, Luo Y, et al. Temperature changes between neighboring days and mortality in summer: a distributed lag non-linear time series analysis. PloS One. 2013 Jun 24;8(6):e66403. doi: 10.1371/journal.pone.0066403 23826095 PMC3691212

[7] Lim YH, Reid CE, Mann JK, Jerrett M, Kim H. Diurnal temperature range and short-term mortality in large US communities. International Journal of Biometeorology. 2015 Sep;59:1311–9. doi: 10.1007/s00484-014-0941-2 25465402

[8] Cheng J, Zhu R, Xu Z, Xu X, Wang X, Li K, et al. Temperature variation between neighboring days and mortality: a distributed lag non-linear analysis. International Journal of Public Health. 2014 Dec;59:923–31. doi: 10.1007/s00038-014-0611-5 25280527

[9] Zhan Z, Zhao Y, Pang S, Zhong X, Wu C, Ding Z. Temperature change between neighboring days and mortality in United States: a nationwide study. Science of the Total Environment. 2017 Apr 15;584:1152–61. doi: 10.1016/j.scitotenv.2017.01.177 28162760

[10] Zhang Y, Peng M, Wang L, Yu C. Association of diurnal temperature range with daily mortality in England and Wales: a nationwide time-series study. Science of the Total Environment. 2018 Apr 1;619:291–300. doi: 10.1016/j.scitotenv.2017.11.056 29154047

[11] Guo Y, Gasparrini A, Armstrong B, Li S, Tawatsupa B, Tobias A, et al. Global variation in the effects of ambient temperature on mortality: a systematic evaluation. Epidemiology (Cambridge, Mass.). 2014 Nov;25(6):781. doi: 10.1097/EDE.0000000000000165 25166878 PMC4180721

[12] Davis RE, Hondula DM, Sharif H. Examining the diurnal temperature range enigma: why is human health related to the daily change in temperature?. International Journal of Biometeorology. 2020 Mar;64:397–407. doi: 10.1007/s00484-019-01825-8 31720855

[13] Guo Y, Gasparrini A, Armstrong BG, Tawatsupa B, Tobias A, Lavigne E, et al. Temperature variability and mortality: a multi-country study. Environmental Health Perspectives. 2016 Oct;124(10):1554–9. doi: 10.1289/EHP149 27258598 PMC5047764

[14] Guo Y, Barnett AG, Yu W, Pan X, Ye X, Huang C, et al. A large change in temperature between neighbouring days increases the risk of mortality. PloS One. 2011 Feb 2;6(2):e16511. doi: 10.1371/journal.pone.0016511 21311772 PMC3032790

[15] Gasparrini A. Distributed Lag Linear and Non-Linear Models in R: The Package dlnm. J Stat Softw. 2011 Jul;43(8):1–20. 22003319 PMC3191524

[16] Wernli H, Schwierz C. Surface cyclones in the ERA-40 dataset (1958–2001). Part I: Novel identification method and global climatology. Journal of the Atmospheric Sciences. 2006 Oct 1;63(10):2486–507. 10.1175/JAS3766.1.

[17] Manney GL, Hegglin MI, Daffer WH, Schwartz MJ, Santee ML, Pawson S. Climatology of upper tropospheric–lower stratospheric (UTLS) jets and tropopauses in MERRA. Journal of Climate. 2014 May 1;27(9):3248–71. doi: 10.1175/JCLI-D-13-00243.1

[18] Burton AC, Edholm OG. Man in a cold environment. Physiological and pathological effects of exposure to low temperatures. Edward Arnold, London; 1955.

[19] De Freitas CR, Grigorieva EA. The impact of acclimatization on thermophysiological strain for contrasting regional climates. International Journal of Biometeorology. 2014 Dec;58:2129–37. doi: 10.1007/s00484-014-0813-9 24633499

[20] Kenny GP, Poirier MP, Metsios GS, Boulay P, Dervis S, Friesen BJ, et al. Hyperthermia and cardiovascular strain during an extreme heat exposure in young versus older adults. Temperature. 2017 Mar 31;4(1):79–88. 10.1080/23328940.2016.1230171 28349096 PMC5356213

[21] Williams CG, Wyndham CH, Morrison JF. Rate of loss of acclimatization in summer and winter. Journal of Applied Physiology. 1967 Jan;22(1):21–6. doi: 10.1152/jappl.1967.22.1.21 6017648

[22] Wyndham CH, Rogers GG, Senay LC, Mitchell D. Acclimization in a hot, humid environment: cardiovascular adjustments. Journal of Applied Physiology. 1976 May 1;40(5):779–85. doi: 10.1152/jappl.1976.40.5.779 931906

[23] Armstrong L.E., Maresh C.M. The Induction and Decay of Heat Acclimatisation in Trained Athletes. Sports Medicine 12, 302–312 (1991). doi: 10.2165/00007256-199112050-00003 1763248

[24] Mathew L, Purkayastha SS, Jayashankar A, Nayar HS. Physiological characteristics of cold acclimatization in man. International Journal of Biometeorology. 1981 Sep;25:191–8. doi: 10.1007/BF02184518 7275347

[25] Höppe P. Temperatures of expired air under varying climatic conditions. Int J Biometeorol. 1981 25:127–132. doi: 10.1007/BF02184460 7251219

[26] Rusanov VI. Appraisal of meteorological conditions defining human respiration. Bull Russ Acad Med Sci. 1989;1:57–60. (in Russian)

[27] Davis RE, Enfield KB. Respiratory hospital admissions and weather changes: a retrospective study in Charlottesville, Virginia, USA. International Journal of Biometeorology. 2018 Jun;62:1015–25. doi: 10.1007/s00484-018-1503-9 29417216

[28] De Freitas CR, Grigorieva EA. Role of acclimatization in weather-related human mortality during the transition seasons of autumn and spring in a thermally extreme mid-latitude continental climate. International Journal of Environmental Research and Public Health. 2015 Dec;12(12):14974–87. 10.3390/ijerph121214962 26703633 PMC4690898

[29] Zhou X, Zhao A, Meng X, Chen R, Kuang X, Duan X, et al. Acute effects of diurnal temperature range on mortality in 8 Chinese cities. Science of the Total Environment. 2014 Sep 15;493:92–7. doi: 10.1016/j.scitotenv.2014.05.116 24937494

[30] Wen B, Wu Y, Guo Y, Li S. A new method to separate the impacts of interday and intraday temperature variability on mortality. BMC Medical Research Methodology. 2023 Apr 15;23(1):92. doi: 10.1186/s12874-023-01914-8 37061686 PMC10105159

