# Supplementary material for: The association between short-term temperature variability and mortality in Virginia
Source: PLoS One. 2024 Sep 20;19(9):e0310545. doi: 10.1371/journal.pone.0310545 (PMC11414919; doi:10.1371/journal.pone.0310545)
Supplement: S1 Table — (DOCX) [file pone.0310545.s001.docx]

**S1 Table. List of counties and independent cities associated with each of the four regions examined: Northern Virginia (IAD), Norfolk (ORF), Richmond (RIC), and Roanoke (ROA).**

| **County** | **Region** |
| --- | --- |
| Alexandria City/County | IAD |
| Alleghany County | ROA |
| Amelia County | RIC |
| Arlington County | IAD |
| Botetourt County | ROA |
| Charles City County | RIC |
| Chesapeake City/County | ORF |
| Chesterfield County | RIC |
| Colonial Heights City/County | RIC |
| Covington City/County | ROA |
| Craig County | ROA |
| Cumberland County | RIC |
| Fairfax City/County | IAD |
| Fairfax County | IAD |
| Falls Church City/County | IAD |
| Fauquier County | IAD |
| Floyd County | ROA |
| Giles County | ROA |
| Goochland County | RIC |
| Hanover County | RIC |
| Henrico County | RIC |
| Hopewell City/County | RIC |
| Isle of Wight County | ORF |
| King and Queen County | RIC |
| King William County | RIC |
| Loudon County | IAD |
| Manassas City/County | IAD |
| Manassas Park City/County | IAD |
| Montgomery County | ROA |
| New Kent County | RIC |
| Norfolk City/County | ORF |
| Petersburg City/County | RIC |
| Portsmouth City/County | ORF |
| Powhatan County | RIC |
| Prince George County | RIC |
| Prince William County | IAD |
| Pulaski County | ROA |
| Radford City/County | ROA |
| Richmond City/County | RIC |
| Roanoke City/County | ROA |
| Roanoke County | ROA |
| Salem City/County | ROA |
| Suffolk City/County | ORF |
| Virginia Beach City/County | ORF |
