# Supplementary material for: The association between short-term temperature variability and mortality in Virginia
Source: PLoS One. 2024 Sep 20;19(9):e0310545. doi: 10.1371/journal.pone.0310545 (PMC11414919; doi:10.1371/journal.pone.0310545)
Supplement: S2 Table — Dof: degrees of freedom, dow: day of week, GCV: generalized cross validation, AIC: Akaike’s Information Criterion. (DOCX) [file pone.0310545.s002.docx]

**S2 Table. Generalized additive model diagnostic information for the maximum and mean temperature difference models.**

| **Station** | **Trend** | **Temp Diff Variable** | **Spline, dof** | **Spline, dof** | **Factor** | **GCV** | **Adj. r^2^** | **Deviance explained** | **Link** | **AIC** |
| --- | --- | --- | --- | --- | --- | --- | --- | --- | --- | --- |
| IAD | 16*3 | Max | PM_2.5_, 3 | Ozone, 3 | dow | 1.0932 | 0.309 | 30.7 | Quasi-Poisson |  |
| IAD | 16*3 | Mean | PM_2.5_, 3 | Ozone, 3 | dow | 1.0952 | 0.307 | 30.5 | Quasi-Poisson |  |
| RIC | 16*3 | Max | PM_2.5_, 3 | Ozone, 3 | dow | 1.0702 | 0.288 | 28.9 | Quasi-Poisson |  |
| RIC | 16*3 | Mean | PM_2.5_, 3 | Ozone, 3 | dow | 1.0739 | 0.285 | 28.6 | Quasi-Poisson |  |
| ORF | 16*3 | Max | PM_2.5_, 3 | Ozone, 3 | dow | 1.0615 | 0.182 | 18.6 | Quasi-Poisson |  |
| ORF | 16*3 | Mean | PM_2.5_, 3 | Ozone, 3 | dow | 1.0629 | 0.181 | 18.5 | Quasi-Poisson |  |
| ROA | 16*3 | Max | PM_2.5_, 3 | Ozone, 3 | dow |  | 0.138 | 14 | Poisson | 31339.81 |
| ROA | 16*3 | Mean | PM_2.5_, 3 | Ozone, 3 | dow |  | 0.137 | 13.9 | Poisson | 31345.83 |

Dof: degrees of freedom, dow: day of week, GCV: generalized cross validation, AIC: Akaike’s Information Criterion
