# Supplementary material for: The association between short-term temperature variability and mortality in Virginia
Source: PLoS One. 2024 Sep 20;19(9):e0310545. doi: 10.1371/journal.pone.0310545 (PMC11414919; doi:10.1371/journal.pone.0310545)
Supplement: S3 Table — The base model is summarized in Eq 1. ns: natural cubic spline, bs: polynomial spline. (DOCX) [file pone.0310545.s003.docx]

**S3 Table. Akaike's Information Criterion (AIC) for Varying Numbers of Equally-spaced Knots and Types of Splines.** The base model is summarized in equation 1. ns: natural cubic spline, bs: polynomial spline.

|  | **Type of Spline** | **n=1** | **n=2** | **n=3** | **n=4** |
| --- | --- | --- | --- | --- | --- |
| **IAD** | ns | 36171 | 36177 | 36175 | 36171 |
| **IAD** | bs | 36171 | 36177 | 36175 | 36171 |
| **RIC** | ns | 35839 | 35845 | 35846 | 35855 |
| **RIC** | bs | 35839 | 35845 | 35846 | 35855 |
| **ORF** | ns | 34939 | 34949 | 34949 | 34952 |
| **ORF** | bs | 34939 | 34949 | 34949 | 34952 |
| **ROA** | ns | 31223 | 31231 | 31231 | 31236 |
| **ROA** | bs | 31223 | 31231 | 31231 | 31236 |

ns: natural cublic spline, bs: polynomial spline
