# Supplementary material for: The association between short-term temperature variability and mortality in Virginia
Source: PLoS One. 2024 Sep 20;19(9):e0310545. doi: 10.1371/journal.pone.0310545 (PMC11414919; doi:10.1371/journal.pone.0310545)
Supplement: S1 Fig — The number at the top of each figure indicates the degrees of freedom per year. (Note that with more than 3 degrees of freedom, there is little change in the overall pattern). (DOCX) [file pone.0310545.s004.docx]

**S1 Figure. Fit of the trend term only for IAD.** The number at the top of each figure indicates the degrees of freedom per year. (Note that with more than 3 degrees of freedom, there is little change in the overall pattern.)

*
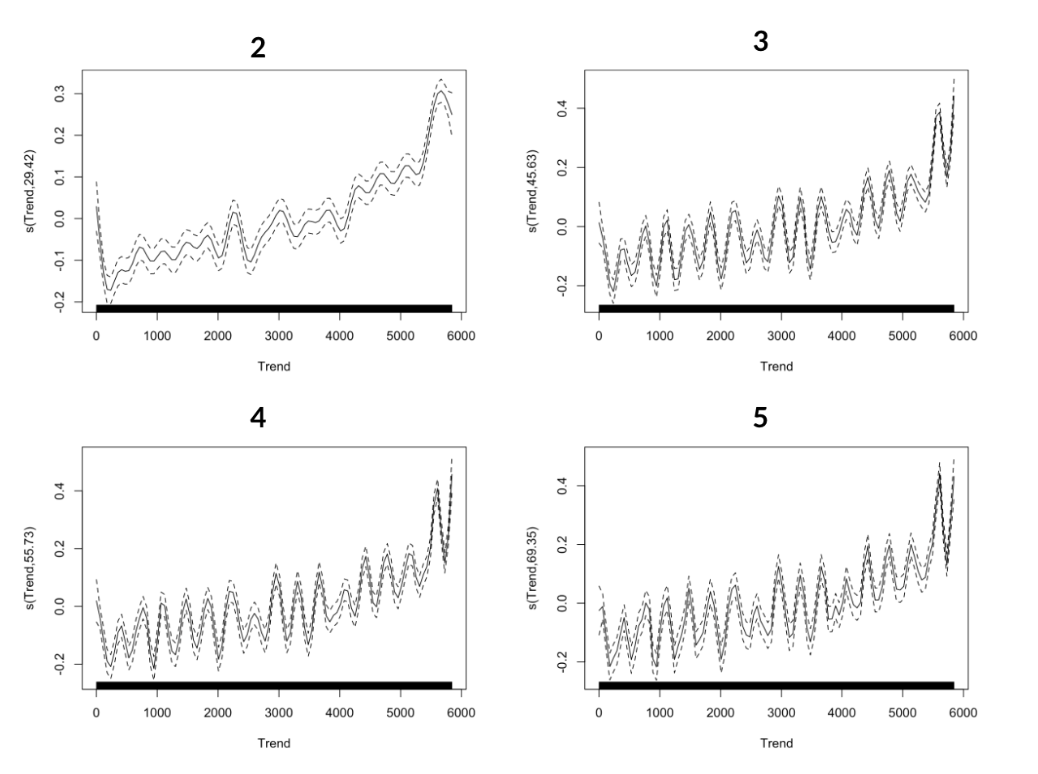
*
