# Supplementary figures and images for: The association between short-term temperature variability and mortality in Virginia
Source: PLoS One. 2024 Sep 20;19(9):e0310545. doi: 10.1371/journal.pone.0310545 (PMC11414919; doi:10.1371/journal.pone.0310545)

**S2 Figure. Comparison of DLNM results for a maximum lag of 21 vs. 28 days at RIC and ORF.**

*
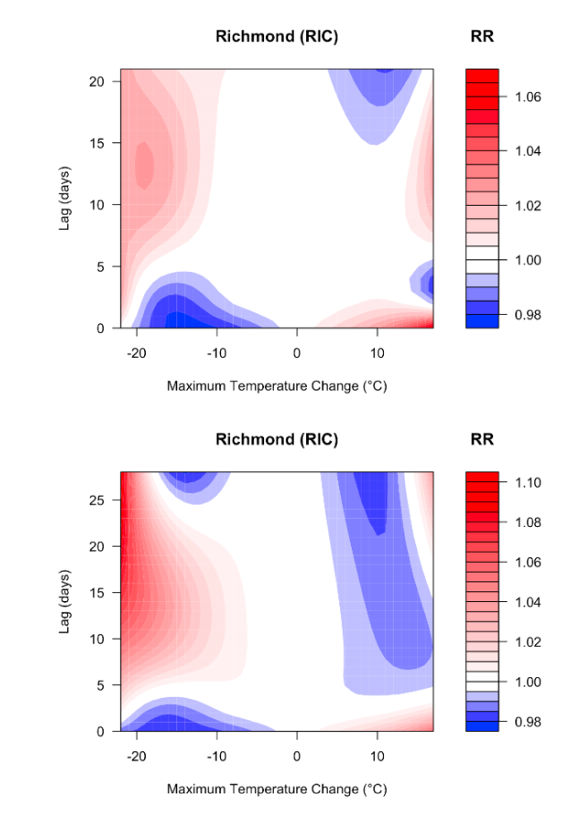

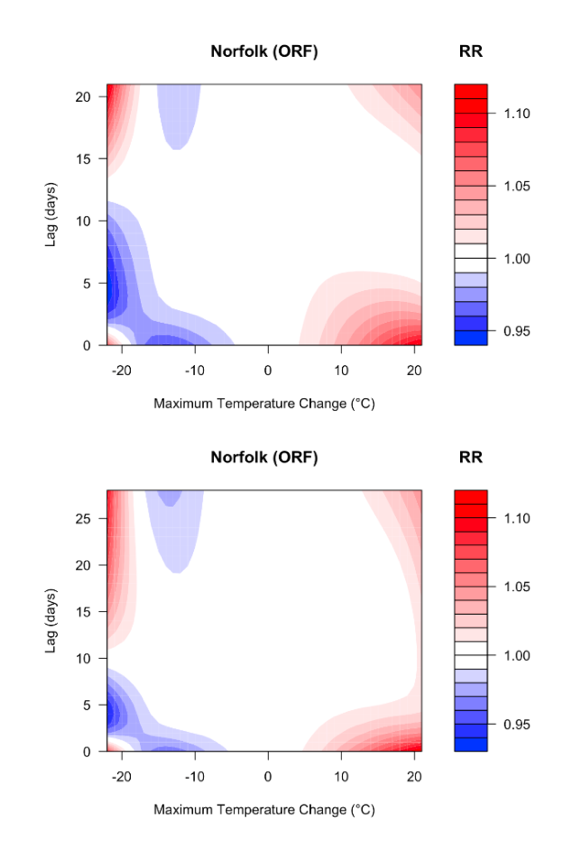
*

Supplement: S2 Fig — (DOCX) [file pone.0310545.s005.docx]
