# Supplementary material for: The association between short-term temperature variability and mortality in Virginia
Source: PLoS One. 2024 Sep 20;19(9):e0310545. doi: 10.1371/journal.pone.0310545 (PMC11414919; doi:10.1371/journal.pone.0310545)
Supplement: S3 Fig — 4 knots (top left), 5 knots (top right), 6 knots (bottom left), 7 knots (bottom right). (DOCX) [file pone.0310545.s006.docx]

**S3 Figure. Impact of number of equally-spaced knots or overall relative risk estimates as a function of maximum temperature change at ROA.** 4 knots (top left), 5 knots (top right), 6 knots (bottom left), 7 knots (bottom right).

*
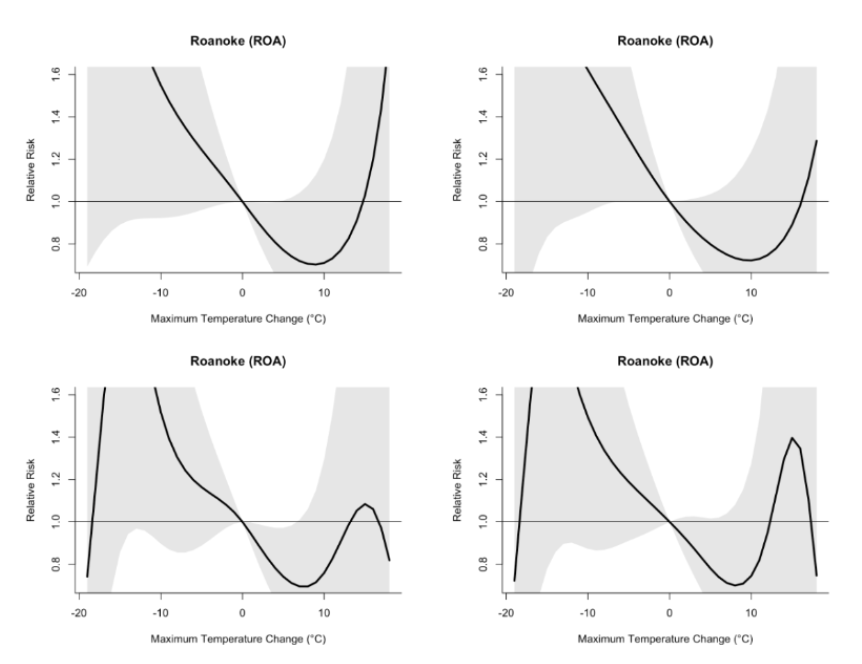
*
